# Expanding surgical access in Africa through improved health insurance schemes: A review

**DOI:** 10.1097/MD.0000000000037488

**Published:** 2024-03-15

**Authors:** Nicholas Aderinto, Gbolahan Olatunji, Emmanuel Kokori, Moradeyo Akanmu Abdulrahmon, Ayodeji Akinmeji, John Oluwasegun Fatoye

**Affiliations:** a Department of Medicine, Ladoke Akintola University of Technology, Ogbomoso, Nigeria; b Department of Medicine and Surgery, University of Ilorin, Ilorin, Nigeria; c Department of Medicine, Olabisi Onabanjo University, Ogun, Nigeria.

**Keywords:** Africa, government policy, health insurance, surgical access

## Abstract

Surgical access remains a pressing public health concern in African nations, with a substantial portion of the population facing challenges in obtaining safe, timely, and affordable surgical care. This paper delves into the impact of health insurance schemes on surgical accessibility in Africa, exploring the barriers, challenges, and future directions. It highlights how high out-of-pocket costs, reliance on traditional healing practices, and inadequate surgical infrastructure hinder surgical utilization. Financing mechanisms often need to be more effective, and health insurance programs face resistance within the informal sector. Additionally, coverage of the poor remains a fundamental challenge, with geographical and accessibility barriers compounding the issue. Government policies, often marked by inconsistency and insufficient allocation of resources, create further obstacles. However, strategic purchasing and fund integration offer avenues for improving the efficiency of health insurance programs. The paper concludes by offering policy recommendations, emphasizing the importance of inclusive policies, streamlined financing mechanisms, coverage expansion, and enhanced strategic purchasing to bridge the surgical access gap in Africa. Decoupling entitlement from the payment of contributions, broadening the scope of coverage for outpatient medicines and related expenses, and enhancing safeguards against overall costs and charges, especially for individuals with lower incomes. Ultimately, by addressing these challenges and harnessing the potential of health insurance schemes, the continent can move closer to achieving universal surgical care and improving the well-being of its people.

## 1. Introduction

In African countries, surgical access remains a persistent and critical public health challenge, where many people grapple with barriers to securing safe, timely, and affordable surgical care. In the global context, surgically treatable diseases contribute 11% to 15% of health impairments, highlighting the growing recognition of surgery as a pivotal facet of global public health.^[1]^ In 2015, the Lancet Commission on Global Surgery estimated that nearly 5 billion people lack access to safe, affordable, timely surgical, and anesthesia care.^[1]^ Nevertheless, despite this substantial burden, many individuals in dire need continue to face insurmountable barriers to surgical services. Sub-Saharan African (SSA) healthcare systems, in particular, often suffer from a lack of focus on surgical conditions, resulting in lower surgical output compared to their high-income counterparts.^[2]^ This discrepancy is acutely felt in West Africa, where the demand for surgical services is particularly high.^[3,4]^

The challenge of surgical access in African countries is multi-faceted, with financial barriers emerging as the foremost obstacle.^[5]^ Surgical procedures frequently incur significant out-of-pocket expenses, rendering it difficult for individuals to access the necessary surgical treatment. These out-of-pocket payments (OOP) reduce healthcare service utilization and lead to catastrophic health expenditures. For instance, OOP constitute more than 70% of healthcare costs in Nigeria, far exceeding the recommended threshold of 30%.^[3,6,7]^ To address this issue, some nations have introduced community-based health insurance plans designed to diminish out-of-pocket expenses and enhance access to healthcare services, particularly for individuals residing in rural areas or those in the informal sector (neither taxed nor monitored by any form of government).

The affordability of surgical procedures poses a unique challenge in low- and middle-income countries, given the cost of healthcare with the average annual household expenditures in many of these nations.^[8,9]^ Furthermore, the field of surgery needs to be more represented, with surgical conditions and care not consistently recognized as paramount to public health.^[10]^ To bolster the resilience of surgical systems, surgery must be prioritized within strategic national health policies. This perspective paper delves into the impact of Health Insurance Schemes on Surgical Accessibility in African countries, shedding light on the potential solutions and challenges such schemes pose in enhancing surgical access and overall public health.

## 2. The role of health insurance in surgical access

SSA countries, including Nigeria, Ethiopia, Tanzania, and Rwanda, have adopted National Surgical, Obstetrics, and Anesthesia Plans to align with the 2030 targets set by the Lancet Commission on Global Surgery, Obstetrics, and Anesthesia.^[11]^ However, despite these efforts, most SSA citizens still allocate a significant portion of their nonfood income to healthcare expenses, leaving them vulnerable to financial hardships and poverty.^[12]^

### 2.1. Financial coverage

In Africa, the financial burden of surgical procedures presents a formidable obstacle to healthcare access. Ninety-three percent of people in SSA cannot secure even the most basic surgical care.^[13]^ This challenge is particularly daunting in a region where 90% of those living in extreme poverty reside.^[11]^ Health insurance, whether publicly funded or privately obtained, can substantially reduce out-of-pocket expenses for surgery.^[14]^ This financial coverage can include the cost of surgeries, hospital stays, medications, and postoperative care.^[14]^ By mitigating the high costs associated with surgical interventions, health insurance ensures that individuals do not have to choose between their health and financial stability.

### 2.2. Safety and availability of surgical services

Health insurance can be a powerful incentive for healthcare providers to offer a wider range of surgical services and increase the number of skilled surgical professionals. When providers know insurance will reimburse surgeries, they are more likely to invest in expanding their surgical capabilities.^[15]^ This can involve upgrading facilities, purchasing advanced medical equipment, and training medical staff. Additionally, insurance can stimulate the recruitment and retention of skilled surgeons, as they are more likely to practice in areas where they can secure service payments.^[15]^ Expanding surgical services and expertise enhances access and elevates the overall healthcare quality available to patients.

### 2.3. Quality of care

Health insurance can improve surgical care in Africa by ensuring access to better-equipped facilities and skilled surgeons. Insurance plans often require providers to meet specific quality standards to participate.^[16]^ As a result, hospitals and clinics can invest in modern equipment, maintain stringent hygiene standards, and employ experienced surgical teams to meet insurance requirements. This focus on quality improvement benefits patients by reducing the risk of complications and ensuring safer surgeries. Access to skilled surgeons and well-equipped facilities also increases the chances of successful outcomes, which is particularly important in complex surgical cases.

### 2.4. Preventative care

Health insurance plays a crucial role in promoting preventative healthcare, which can reduce the need for surgical interventions.^[17]^ Insurance plans often cover preventive services such as vaccinations, screenings, and regular checkups.^[18]^ By providing these services at little or no cost to individuals, insurance encourages people to seek early medical attention, leading to the detection, and management of health issues before they escalate to the point where surgery is required. Preventative care can address conditions like diabetes, hypertension, and certain cancers, reducing the overall demand for surgical procedures and improving the population’s health.

## 3. Barriers and challenges

Implementing health insurance schemes in Africa encounters various obstacles that span different aspects of the healthcare system. These challenges hinder the effective rollout and utilization of health insurance programs for improving surgical access.

### 3.1. Health service utilization

Efforts to enhance health insurance for improved surgical access in Africa encounter multifaceted challenges concerning health service utilization. High out-of-pocket costs at government healthcare facilities render surgical care financially inaccessible for many individuals.^[19]^ In addition, the influence of traditional healing practices poses a significant challenge. Many African communities place deep-rooted trust in these traditional methods, diverting patients from modern surgical interventions.^[20]^ This shift in preference affects the utilization of the available health insurance and perpetuates the underutilization of surgical services despite their potential to provide safer and more effective treatments.

### 3.2. Financing mechanisms

Effective financing mechanisms are essential for the success of health insurance programs to improve surgical access in Africa. In many cases, ineffective pooling systems fail to collect and manage funds for healthcare services efficiently, hindering the stability and sustainability of health insurance programs.^[15]^ Moreover, the coexistence of formal and informal sectors in African economies poses unique health insurance and surgical access challenges.^[21]^ The large informal sector is characterized by unstable employment and income, making it challenging for individuals to afford health insurance.^[21]^ Moreover, health insurance is not readily accepted by informal sector workers due to various barriers, including affordability and a need for more awareness regarding its benefits.

### 3.3. Coverage of the poor

Ensuring that health insurance programs cover the most vulnerable populations, particularly the poor, is a fundamental challenge in Africa. Many programs rely on voluntary enrollment, leaving a substantial portion of the population needing more coverage.^[22]^ Furthermore, there is often an urban-centric focus, with rural and underserved areas receiving less attention and fewer resources.^[22]^ Geographical barriers, such as the distribution of healthcare facilities, can create access challenges, particularly for rural populations.^[23]^ The inaccessibility of healthcare facilities in remote areas further hampers the utilization of health insurance for surgical care.

### 3.4. Government policies

Government policies significantly influence the healthcare landscape and the overall success of health insurance programs. In Africa, most policies inadvertently marginalize the economically disadvantaged and create substantial barriers to accessing health insurance and, by extension, surgical care.^[24]^ Such policies hinder the populations health insurance programs aim to serve, perpetuating health disparities, and inequities. Inconsistencies in health insurance and healthcare delivery policies further exacerbate challenges. The lack of a clear and uniform policy framework needs to be clarified for providers and beneficiaries. This confusion must be addressed to ensure effective program implementation, reducing the overall impact of health insurance on surgical access. More allocation of resources to healthcare constitutes another policy-related challenge. When healthcare funding falls short of the actual need, health insurance programs need help to cover surgical intervention costs adequately. Rwanda exhibits the most favorable level of OOP, with a modest rate of 6.3%.^[25]^ In contrast, other countries such as Ghana, Ethiopia, Nigeria, and Sudan have significantly higher OOP rates, exceeding 35%.^[25]^ This contradicts the guideline set forth by the World Health Organization, which advises that the proportion of total OOP should be at most 15% to 20% of a country’s national health expenditures.^[26]^ This funding gap limits the ability of health insurance programs to provide comprehensive surgical access, leaving many individuals needing more care. Additionally, poor decentralization within the healthcare administration significantly affects the efficient delivery of surgical services to those in need.

## 4. Future directions and policy recommendations

As the importance of health insurance in improving surgical access in Africa becomes increasingly evident, it is essential to chart future directions and provide policy recommendations that can enhance the effectiveness of these programs.

### 4.1. Expanding health insurance coverage

The key to improving surgical access in Africa lies in expanding health insurance coverage to reach universal health coverage. Policymakers must prioritize and allocate resources to scale up existing public health insurance programs, such as the National Health Insurance Schemes and Community-Based Health Insurance plans. These programs can serve as the foundation for extending coverage to a broader population. Mandatory health insurance can be introduced to achieve universal health coverage, ensuring every citizen can access surgical care. This approach spreads the financial risk across a larger pool and encourages wealthier individuals to participate, thereby subsidizing the insurance of those with lower incomes. Subsidies and targeted financial support should be provided to the poorest sections of the population to guarantee that health insurance remains affordable. Moreover, it is crucial to underscore the imperative of targeted education for segments of society below the poverty line, especially within the informal sectors. The prevailing disparities in healthcare access are exacerbated in these vulnerable communities due to a lack of awareness and limited resources. Therefore, an enhanced commitment to educating and empowering individuals in informal sectors becomes paramount. Concurrently, recognizing the essential role of health insurance, it is imperative to broaden the scope of public health campaigns. These campaigns should not only emphasize the importance of health insurance but also specifically target urban areas while extending their reach to remote and underserved regions.

### 4.2. Tailored insurance programs

One-size-fits-all health insurance programs may not effectively address the unique needs of the African population. Policymakers should design tailored insurance programs that consider the irregular income patterns of informal sector workers, who often earn income sporadically and may not afford regular premium payments. Additionally, geographical disparities in surgical access must be addressed. Programs should be developed to make surgical services available in remote areas. Mobile health units and telemedicine can play a significant role in reaching individuals in underserved regions. Innovative transportation solutions can be employed to ensure patients can access surgical care without incurring excessive travel costs or facing logistical difficulties.

### 4.3. Enhancing financing mechanisms

Efficient financing mechanisms are essential for the sustainability of health insurance programs. Policymakers should focus on optimizing premium collection to ensure that premiums are collected on time and in a manner that is convenient for beneficiaries. This could involve leveraging digital payment platforms, mobile banking, or community-based agents who can facilitate payments. Risk-pooling mechanisms should be enhanced to distribute financial risks across the insured population. Developing innovative financial instruments, such as catastrophe bonds, can help manage the costs associated with major health crises or surgical emergencies. These bonds can provide additional funding during times of crisis, ensuring that health insurance programs remain resilient. Reforms in taxation can also play a significant role in supporting health insurance programs. A portion of taxes on goods and services can be allocated to health insurance to create a stable funding source. Policymakers should collaborate with tax authorities to ensure the efficient collection and allocation of these funds.

### 4.4. Leveraging digital health solutions

Digital health solutions have the potential to revolutionize health insurance programs in Africa. Policymakers should prioritize integrating digital technology to enhance the convenience and accessibility of health insurance. Mobile technology can be harnessed for premium payments, claims processing, and appointment scheduling. Mobile banking and digital wallets can make it easier for beneficiaries to pay their premiums, reducing the barriers associated with traditional payment methods. Patients can also use mobile apps or short message service services to schedule appointments and access important surgical information. Moreover, digital platforms can improve the overall management of health insurance programs. They enable real-time tracking of premiums, claims, and surgical care service utilization. This data can inform policy decisions, identify areas for improvement, and ensure the efficient allocation of resources.

### 4.5. Investment in healthcare infrastructure

Strengthening domestic surgical care infrastructure is critical for improving surgical access. African countries should prioritize investment in surgical facilities and the training of surgeons. Improving the availability and quality of surgical services is essential. This includes upgrading facilities, ensuring the availability of essential surgical equipment, and maintaining stringent hygiene standards. Investment in specialized surgical units and trauma centers can ensure that critical surgical care is readily available. Training and retaining skilled surgeons and healthcare professionals is crucial. Programs to support medical education and professional development can help build a robust surgical workforce. This investment increases the availability of skilled personnel and enhances the overall quality of healthcare delivery. Efforts should be made to reduce the reliance on surgical tourism by improving domestic healthcare infrastructure. To build trust in local healthcare systems, policies should focus on bolstering healthcare facilities, equipment, and expertise. This keeps resources within the country and makes surgical care more appealing for patients, reducing the need to seek treatment abroad.

### 4.6. Monitoring progress and evaluating outcomes

Embarking on the initiative to enhance surgical access in Africa through improved health insurance schemes necessitates the establishment of comprehensive metrics for progress monitoring and outcome evaluation. These metrics will serve as navigational tools for gauging the effectiveness of the implemented reforms and as sources of critical insights for ministries of health to refine financial incentives and optimize resource allocation. Tracking surgical procedure rates is foundational. This involves monitoring the number of surgical procedures performed post-implementation, examining the distribution across different regions and demographic groups, and ensuring equitable access. Similarly, evaluating the increase in health insurance enrollment and participation rates is crucial to understanding the extent of acceptance within the target population. The geographical accessibility of surgical facilities is another pivotal metric. Analyzing the distribution and proximity of these facilities to population centers will illuminate whether the reforms effectively address regional disparities and bring surgical services closer to remote and underserved areas. Complementing these quantitative metrics, it is imperative to assess the financial impact on patients, comparing the burden before and after the implementation of health insurance schemes to understand their efficacy in reducing out-of-pocket expenses and enhancing financial accessibility to surgical care. Similarly, monitoring postsurgical recovery rates, complication rates, and overall patient satisfaction to gain insights into the qualitative impact of the program. Armed with data from these metrics and outcomes, the relevant stakeholders can proactively refine financial incentives, fine-tune policy interventions, and adapt strategies to address emerging challenges. Regular evaluations ensure a dynamic and responsive approach to healthcare reforms, facilitating sustained progress in expanding surgical access across Africa.

## 5. Conclusion

The pressing need for improved surgical access in Africa has brought health insurance schemes to the forefront as a potential solution. The challenges and barriers to achieving this goal are multifaceted, ranging from financial and policy inconsistencies to organizational limitations and cultural factors. However, the potential benefits are equally profound, with health insurance programs offering opportunities to alleviate financial burdens, enhance service quality, and expand surgical services. To move forward, African nations must consider a comprehensive approach that addresses the identified challenges and leverages the strengths of health insurance schemes. Health insurance can play a pivotal role in closing the surgical access gap in Africa by crafting inclusive policies, streamlining financing mechanisms, ensuring coverage of vulnerable populations, and enhancing strategic purchasing. Through these concerted efforts and a commitment to equitable healthcare, the vision of universal surgical access can be realized, improving the lives and well-being of countless individuals across the continent.

## Author contributions

**Conceptualization:** Nicholas Aderinto.**Data curation:** Nicholas Aderinto, Gbolahan Olatunji and Emmanuel Kokori.**Formal analysis:** Nicholas Aderinto, Gbolahan Olatunji and Emmanuel Kokori.

**Writing – original draft:** Nicholas Aderinto, Gbolahan Olatunji, Emmanuel Kokori, Moradeyo Akanmu Abdulrahmon, Ayodeji Akinmeji, John Oluwasegun Fatoye.

**Writing – review & editing:** Nicholas Aderinto, Gbolahan Olatunji, Emmanuel Kokori, Moradeyo Akanmu Abdulrahmon, Ayodeji Akinmeji, John Oluwasegun Fatoye.
